# A Novel Anti-Noise Fault Diagnosis Approach for Rolling Bearings Based on Convolutional Neural Network Fusing Frequency Domain Feature Matching Algorithm

**DOI:** 10.3390/s21165532

**Published:** 2021-08-17

**Authors:** Xiangyu Zhou, Shanjun Mao, Mei Li

**Affiliations:** Institute of Remote Sensing and Geographic Information System, Peking University, Beijing 100871, China; zxy0112k@pku.edu.cn (X.Z.); mli@pku.edu.cn (M.L.)

**Keywords:** fault diagnosis, convolutional neural network, deep learning, anti-noise

## Abstract

The development of deep learning provides a new research method for fault diagnosis. However, in the industrial field, the labeled samples are insufficient and the noise interference is strong so that raw data obtained by the sensor are occupied with noise signal. It is difficult to recognize time-domain fault signals under the severe noise environment. In order to solve these problems, the convolutional neural network (CNN) fusing frequency domain feature matching algorithm (FDFM), called CNN-FDFM, is proposed in this paper. FDFM extracts key frequency features from signals in the frequency domain, which can maintain high accuracy in the case of strong noise and limited samples. CNN automatically extracts features from time-domain signals, and by using dropout to simulate noise input and increasing the size of the first-layer convolutional kernel, the anti-noise ability of the network is improved. Softmax with temperature parameter T and D-S evidence theory are used to fuse the two models. As FDFM and CNN can provide different diagnostic information in frequency domain, and time domain, respectively, the fused model CNN-FDFM achieves higher accuracy under severe noise environment. In the experiment, when a signal-to-noise ratio (SNR) drops to -10 dB, the diagnosis accuracy of CNN-FDFM still reaches 93.33%, higher than CNN’s accuracy of 45.43%. Besides, when SNR is greater than -6 dB, the accuracy of CNN-FDFM is higher than 99%.

## 1. Introduction

Along with the rapid development of the modern industry and sensor monitoring technology, a large amount of sensor data can be obtained [1]. Mining valuable information contained in these data is a significant task of intelligent fault diagnosis, which is a current hot spot for scholars [2]. Rotating machinery is widely used in industrial applications, and rolling bearing, as the core component of rotating machinery, is the most vulnerable part though [3]. Bearing failure caused by operation in complex and harsh environment will lead to shutdown of large rotating machinery, which could result in enormous economic loss and even threaten the safety of stuff [4]. Accurate and effective fault diagnosis of rolling bearings, not only reduce the cost of maintenance, but also improve the reliability and stability of the equipment [5].

Generally speaking, we mostly use the vibration signals collected by the sensor as the basis of fault diagnosis [6]. Common intelligent fault diagnosis is mainly constructed by the algorithms of signal processing and pattern recognition. Signal processing techniques extract and select key features from the collected raw vibration signals that contain both useful information and useless noise [7]. Commonly used methods are wavelet analysis [8,9], fourier spectral analysis [10], empirical mode decomposition (EMD) [11,12] and other feature transformation techniques [13,14,15]. However, exquisite technology and rich expert experience are required in the above approaches [16]. Pattern recognition is to identify the fault information within the extracted features by artificial intelligence method and realize automatic fault diagnosis. Machine learning algorithms have been successfully applied in fault diagnosis, such as artificial neural networks (ANN) [17], support vector machine (SVM) [18], k-nearest neighbor (KNN) [19] and hidden Markov model (HMM) [20].

In recent years, with the popularity of deep learning as a computational framework in various research fields, deep learning provides a new research direction for fault diagnosis [21]. Deep learning methods have recently been applied and have realized remarkable results, such as convolutional neural network (CNN) [22,23], recurrent neural network (RNN) [24], deep belief network (DBN) [25], stacked auto-encoders (SAE) [1,26], long short-term memory (LSTM) [27,28].

Many of the works mentioned above have achieved pretty good results, nevertheless, the following problems in industrial sites still need to be considered: (1) Strong noise interfere. It is necessary to study the anti-noise ability of the model due to the strong noise interference in industrial site. (2) Limited labeled samples. The number of fault samples is limited in the industry, which can easily cause over-fitting. We need to extract the key information which can reflect the fault characteristics from the limited samples.

To solve the first problem, Zhang et al. [29] proposed a deep CNN, in which small mini-batch training and kernel dropout were used as interference to simulate the influence of noise. Shi et al. [30] proposes a residual dilated pyramid network combined with full convolutional denoising auto-encoder, which is suitable for different speeds and noise modes. Liu et al. [31] combined a one-dimensional denoising convolutional auto-encoder (DCAE) and a one-dimensional convolutional neural network (CNN) to solve this problem, whereby the former is used for noise reduction of raw vibration signals and the latter for fault diagnosis using the denoised signals. Most of these denoising methods are only applicable to the noisy environment where signal to noise ratio (SNR) is greater than −4 dB, but cannot be applied to more severe noise environment.

To solve the second problem, Zou et al. [5] proposed an adversarial denoising convo- lutional neural network(ADCNN), in which adversarial training was used to expand the labeled samples. This method improved the robustness and generalization of ADCNN, and avoid over-fitting with limited number of labeled samples. Dong et al. [32] proposed a dynamic model of bearing to generate massive and various simulation data, and diagnosis for real scenario are based on transfer strategies and CNN. Pan et al. [33] proposed a semi-supervised multi-scale convolutional generative adversarial network for bearing fault identification when the labeled data are insufficient. These methods mostly generate their own datasets by adversarial training or simulation when the labeled samples are limited.

In addition, when the vibration signal is selected as the original data, the input data can be divided into time domain and frequency domain. Many current application of deep learning models complete feature extraction and classification in one single domain [34]. For the signals in time domain, the characteristics of the fault are not obvious and easily affected by noise. However, for the signals in the frequency domain, different faults have obvious peaks in different frequency bands in the frequency spectrum, and these peaks are still obvious in the case of strong noise. Moreover, the fault characteristics which are not obvious in time domain can be obtained after the signal is converted into frequency domain. The same raw signal can provide different fault information in time domain and frequency domain [35]. The fused fault information is more comprehensive, which can improve the overall accuracy of the model.

In this paper, CNN fusing Frequency Domain Feature Matching algorithm (FDFM) named CNN-FDFM, is proposed to solve the problems of strong noise interference and limited samples in industry field. Compared with previous studies, our model is qualified for severe noise environment with SNR of −10 dB. When solving the problem of limited samples, FDFM focuses on the key features of limited data, which can be used to characterize different fault types, instead of using the method of expanding the data set.

(1) For signals in the frequency domain, the FDFM proposed in this paper can ensure high recognition rate of test samples in strong noise environment, and is also effective when the number of training samples is small.

(2) For signals in the time domain, one-dimensional CNN is used to learn features and complete classification automatically. The trick of dropout acts on the input layer during training, which can simulate the noise input and enhance the anti-noise performance of the network.

(3) By fusing the diagnosis result of the two algorithms with softmax and D-S evidence theory, the information fusion between frequency domain and time domain is realized. Model fusion makes the two algorithms complementary. CNN-FDFM achieves higher diagnosis accuracy and better anti-noise performance. The feasibility and superiority of the model are verified in the experimental data set.

## 2. A Brief Theoretical Background

### 2.1. FFT

Fast Fourier transform (FFT) is an algorithm of discrete Fourier transform (DFT) with efficient and fast computation, which is very important in the field of signal processing. Fourier transform can transform a signal from time domain to frequency domain. The DFT of discrete signals with finite length X(n), n=0,1,2,…, N−1 is defined as:

(1) $$X_{k}=\overset{N-1}{\underset{n=0}{\sum }}x_{n}e^{-i2\pi k\frac{n}{N}}\;,\;k=0,1,2,\ldots ,\;N-1$$

The sampling theory needs to be satisfied when FFT algorithm is carried out, which demands that the sampling frequency $f_{s.max}$ must be greater than two times the highest frequency $f_{max}$ in the signal ($f_{s.max}>2f_{max}$). Therefore, spectral aliasing can be avoided.

Additionally, when the time-domain signal is transformed by FFT, the range of frequency for analysis is determined by the sampling frequency $f_{s.max}$ no matter how many points (the value of N) are taken. If we take N points for FFT, the frequency interval between two adjacent points after the transformation is $f_{s.max}/N$. The frequency of k-th point is $k\times (f_{s.max}/N)$, k=0,1,2,…, N−1. The values of these N points are symmetric, so only N/2 points are actually used. In order to improve the resolution of the spectrum with constant sampling frequency, the length of the sampling data should be extended so that the influence of spectrum leakage can be indirectly reduced.

### 2.2. CNN

As an important method of deep learning, CNN has good effects in speech and image processing. CNN is constructed by three types of layers, which are the convolutional layer, the pooling layer and the fully connected layer. Feature extraction of input data is achieved by the convolutional layer and the pooling layer, while the fully connected layer is mainly responsible for classification.

The input signal is convoluted in the convolutional layer with a series of kernels. Each kernel is used to extract the features from the local input signal. By sliding the kernel with a constant stride and repeating the convolution operation on the data in the new receptive field, the feature of the input signal extracted by one kernel is obtained. The weight of kernel is shared during this this process. The corresponding feature map for each kernel can be obtained by activation function. The process of convolution is described as follows:

(2) $$x_{l}^{i}=f\left(x_{l-1}^{r}*K_{l}^{i}+b_{l}^{i}\right)=f\left(\underset{r}{\sum }x_{l-1}^{r}*K_{l,r}^{i}+b_{l}^{i}\right)$$

where $x_{l}^{i}$ is the i-th output feature map of convolutional layer l; *f* (∙) is a nonlinear activation function; $x_{l-1}^{r}$ is the *r*-th convolutional region of feature map generated from the layer l−1; $K_{l}^{i}$ is weight matrix of i-th kernel in convolutional layer l; $b_{l}^{i}$ is the bias. In CNN, Rectified Linear Unit (ReLu) is commonly used as activation unit to enhance the representation ability. The expression of Relu function is as follows:

(3) $$x_{l}^{i}=\underset{}{\max}\left(0,x_{l}^{i^{\prime }}\right)$$

where $x_{l}^{i}{}^{\prime }$ is the output of i-th kernel in convolutional layer l without nonlinear activation.

Generally, the pooling layer is added to each convolutional layer for generating lower-dimension feature maps by sub-sampling operation. Max-pooling layer is the most commonly used type, which takes the maximum value of the feature in the receptive field as the output. The expression of the max-pooling transformation is as follows:

(4) $$x_{l+1}^{i}=\underset{\left(k-1\right)W+1\leq s\leq kW}{\max}x_{l}^{i}\left(s\right)$$

where $x_{l+1}^{i}$ is the output of the max-pooling layer, $x_{l}^{i}\left(s\right)$ denotes the s-th value in each pooling area, s∈[(k−1)W+1,kW], W is the width of the pooling area.

To integrate and classify the local features extracted from prior layers, the fully connected layer is finally applied. Logits are the output of the fully connected layer. Then, softmax is mainly used in the last layer to transform logits into possibilities, and it can be expressed as follows:

(5) $$P\left(y=i\right)=\mathrm{Softmax}\left(i\right)=\frac{e^{a_{i}}}{\sum_{j=1}^{C}e^{a_{j}}}$$

where P(y=i) is the possibility of the i-th categories (1≤i≤C), C is the number of categories, $a_{i}$ is the i-th value of logits.

## 3. Proposed Fault Diagnosis Method

**Figure 1:**
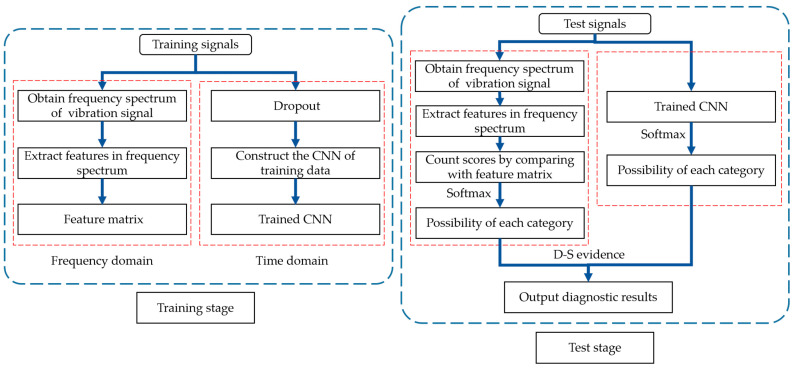
Structure of fault diagnosis method proposed in this paper.

Generally, in order to ensure the generalization of the model, we need plenty of labeled fault samples to train the model. Actually, labeled fault samples are difficult to obtain in an industry field, which could easily cause over-fitting and poor generalization of the model. Additionally, industrial environment is harsh and terrible, covered with a lot of interference, so the data obtained by the sensor are occupied with strong noise. To solve the problems above, we utilize FFT to obtain key frequency features to improve the diagnosis accuracy of CNN under noisy environment as well as in the case of few labeled samples. The principle of feature selection from frequency spectrum, the structure of CNN and the strategies for model fusion are introduced orderly in this part. The structure of fault diagnosis method proposed in this paper is shown in Figure 1.

### 3.1. Frequency Domain Feature Matching Algorithm

Section 2.1 introduces that FFT can transform a signal from time domain to frequency domain. By this means, when time-domain signals are transformed to a frequency domain, the characteristics of the signals can be observed more clearly.

The frequency-domain signal is less affected by noise than the time-domain signal. After the fault signal is converted from time domain to frequency domain, the abscissa corresponding to the peak value in spectrum can be used as the feature frequency of each fault signal. If the working condition remains the same, the noise interference will only change the amplitude of the original frequency, but will not change the location of the original frequency, which means that the abscissa of the peak will not change in the strong noisy environment. The abscissa of the peak in spectrum can represent the feature frequency of the fault in this case.

For some fault types, the segmentation of samples and the interference of noise will cause fluctuation in amplitude, and when noise interference is severe enough, the original peak value will be exceeded by the amplitude of other frequencies. Therefore, in order to ensure that the key features are not lost, we extract a series of feature frequencies according to the descending order of peak value, which constitutes a set of feature frequencies of fault samples. In this paper, the feature sequence generated by each training and test sample is composed of 10 feature frequencies.

**Figure 2:**
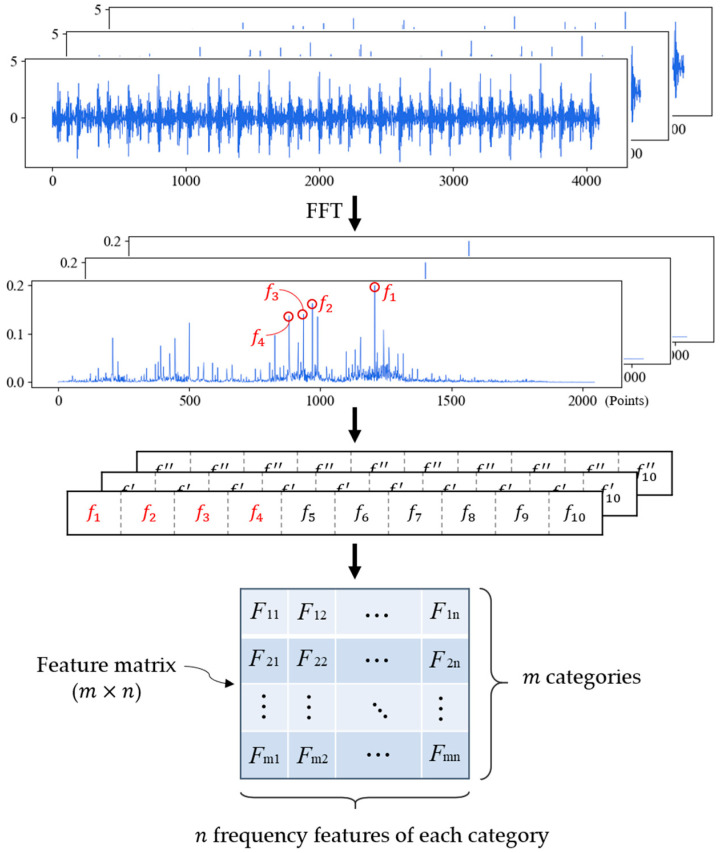
Training stage of FDFM algorithm.

For the samples of the same fault type, we count the occurrence times of feature frequencies in all feature sequences and sort them in descending order. The first n feature frequencies are selected as the feature sequence of this fault type. If there are m fault types, the feature matrix with size of m×n will be generated, which is the final result in training phase. The training process of FDFM is shown in Figure 2.

In test phase, the feature sequence of each test sample is matched with each row of the feature matrix to earn the score. The score is used to measure the matching degree of each category, and the category with the highest score is the final diagnosis result. In order to make the discrimination of samples more obvious, the following three scoring rules are proposed. For h∈[1,m],

(1) Count the number in $\left\{f_{1},f_{2},\;\ldots ,f_{10}\right\}\cap^{\;}\left\{F_{h1},F_{h2},\ldots ,F_{hn}\right\}$, and score 1 point for each number in common.
(2) Count the number in $\left\{f_{1},f_{1}\pm 1,f_{2},\;f_{2}\pm 1,\ldots ,f_{6},f_{6}\pm 1\right\}\cap^{\;}\left\{F_{h1},F_{h2},\ldots ,F_{hn}\right\}$, and score 1 point for each number in common.
(3) Count the number in $\left\{f_{1},f_{2},\;f_{3}\right\}\cap^{\;}\left\{F_{h1},F_{h2},F_{h3}\right\}$, and score 4 point for each number in common.

Where $\left\{f_{1},f_{2},\;\ldots ,f_{10}\right\}$ denotes 10 feature frequencies of each test sample, $\left\{F_{h1},F_{h2},\ldots ,F_{hn}\right\}$ denotes the feature sequence of on the h-th row of the feature matrix. The general procedure of the proposed FDFM algorithm is given in Algorithm 1.
**Algorithm 1****Frequency Domain Feature Matching Algorithm****Input:**Training dataset: $D_{train}=\left\{\left(X_{train}\left(i\right),Y_{train}\left(i\right)\right),i=1,2,3,\cdots ,k\right\}$; length of training dataset: k; Test dataset: $D_{test}=\left\{\left(X_{test}\left(i\right),Y_{test}\left(i\right)\right),i=1,2,3,\cdots ,s\right\}$; length of test dataset: s; Fast Fourier transform (FFT): ℱ(·); The number of selected feature frequencies for each sample: FN=10; The function that returns the index of the array sorted in ascending order: argsort(·); The function that reverses the array and returns the first FN elements:$\;Reverse^{FN}\left(\cdot \right)$; The number of categories: m; The number of selected feature frequencies for each category: n; Scoring function with scoring rules 1, 2 and 3: SR{A,B}; A is feature frequencies; B is feature matrix.**Output:**Feature matrix with size of m×n; Scoreboard of all test samples.**Training stage:****Obtain feature matrix with size of** m×n
**for** i∈[1,k] **do**      $X_{trainFFT}\left(i\right)=ℱ\left(X_{train}\left(i\right)\right)\;$ (Obtain the frequency spectrums of k training samples by FFT);      $\left[f_{1}^{i},f_{2}^{i},\cdots ,f_{FN}^{i}\right]=Reverse^{FN}\left\{argsort\left[X_{trainFFT}\left(i\right)\right]\right\}$;      $Feature\;Frequencies_{\left(i\right)}=\left[f_{1}^{i},f_{2}^{i},\cdots ,f_{FN}^{i}\right]$
      (Extract FN feature frequencies from frequency spectrum of each training sample); **end for** **for** label∈[1,m] **do**      $AF_{label}=\left[\;\right]$;      **for** i∈[1,k] **do**          **if**
$Y_{train}\left(i\right)==label$ **then**          Append $Feature\;Frequencies_{\left(i\right)}$ to the end of the list $AF_{label}$; **end if** **end for**      Count the occurrence times of feature frequencies in $AF_{label}$ and sort them in descending order;      The feature sequence $F_{label}$ consists of the first n feature frequencies;      $F_{label}=\left[F_{label1},F_{label2},\cdots ,F_{labeln}\right]$; **end for** $\mathrm{Feature}\;\mathrm{Matrix}=\left[\begin{array}{cc} \begin{array}{cc} F_{11} & F_{12}\\ F_{21} & F_{22} \end{array} & \begin{array}{cc} \cdots & F_{1n}\\ \cdots & F_{2n} \end{array}\\ \begin{array}{cc} \vdots & \vdots \\ F_{m1} & F_{m2} \end{array} & \begin{array}{cc} \ddots & \vdots \\ \cdots & F_{mn} \end{array} \end{array}\right]$; **return**  Feature Matrix
**Test stage:****Calculate scoreboard of all test samples**
Scoreboard=[  ]; **for** j∈[1,s] **do**      $X_{testFFT}\left(j\right)=ℱ\left(X_{test}\left(j\right)\right)\;$ (Obtain the frequency spectrums of s test samples by FFT);      $\left[f_{1}^{j},f_{2}^{j},\cdots ,f_{FN}^{j}\right]=Reverse^{FN}\left\{argsort\left[X_{testFFT}\left(j\right)\right]\right\}$;      $Feature\;Frequencies_{\left(j\right)}=\left[f_{1}^{j},f_{2}^{j},\cdots ,f_{FN}^{j}\right]$
      (Extract FN feature frequencies from frequency spectrum of each test sample);      $Score_{j}=0$ (Initialize score);      $\left[S_{j1},S_{j2},\cdots ,S_{jm}\right]=$ $SR\left\{Feature\;Frequencies_{\left(j\right)},\;\mathrm{Feature}\;\mathrm{Matrix}\right\}$;      $Score_{j}=\left[S_{j1},S_{j2},\cdots ,S_{jm}\right]$;      Append $Score_{j}$ to the end of the list Scoreboard; **end for** $Scoreboard=\left[\begin{array}{cc} \begin{array}{cc} S_{11} & S_{12}\\ S_{21} & S_{22} \end{array} & \begin{array}{cc} \cdots & S_{1m}\\ \cdots & S_{2m} \end{array}\\ \begin{array}{cc} \vdots & \vdots \\ S_{s1} & S_{s2} \end{array} & \begin{array}{cc} \ddots & \vdots \\ \cdots & S_{sm} \end{array} \end{array}\right]$; **return**  Scoreboard

### 3.2. 1D-CNN with Dropout in the First Layer

**Figure 3:**
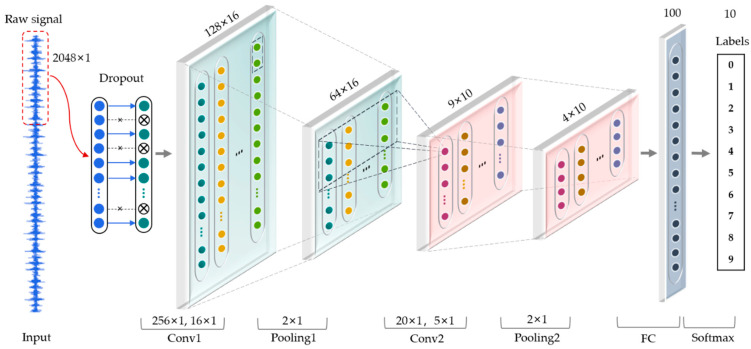
The architecture of one-dimensional convolutional neural network in this paper.

As shown in Figure 3, a one-dimensional convolutional neural network is used to learn features adaptively from raw vibration signal in time domain without prior knowledge. The input of the CNN is a segment of normalized bearing fault vibration temporal signal and dropout is used in the input layer.

Dropout is a trick proposed by Srivastava et al. [36] to prevent the network from overfitting. It is based on the premise that the neural network unit is temporarily deactivated according to a certain probability called dropout rate during training. While in the test phase, dropout is no longer applied. It is found that a network trained with dropout usually leads to much better generalization ability compared to another network trained with other regularization methods. However, in CNN, a dropout is only used for the fully connected layer, but not for other layers. This is because overfitting is not really a problem for convolutional layers which do not have many parameters. The convolutional layers usually use batch normalization as an alternative. In addition to regularization, batch normalization also avoids the problem of gradient disappearance during training of CNN, which can reduce the training time and get better results.

In this paper, dropout is used in the input layer to simulate the noise input during training, which can increase the robustness and anti-noise ability of the network. When the dropout rate of input layer is set to 0.5, samples randomly generated by dropout can achieve the highest diversity.

According to Zhang et al. [29], the wide kernels in the first convolutional layer can better suppress high frequency noise compared with small kernels. In this paper, the kernel size of the first convolutional layer is increased to 256 to obtain the global characteristics of the signal in the longer time domain and reduce the influence of noisy details in the shorter time domain. The detailed parameters of CNN are shown in Table 1.

### 3.3. Fusion Strategies: Softmax with Parameter T and D-S Evidence Theory

According to Section 3.1 and Section 3.2, we can obtain the scoreboard from FDFM algorithm and the output after softmax from CNN, which can be regarded as the normalized probabilities.

Prior to fusing the diagnosis results in the frequency domain and time domain, it is necessary to ensure that the output formats of the two algorithms are consistent, that is, the output should be converted into the probability of each category and the probability distribution is smoothed. First, we need to transform the integer scoreboard into probability distribution. Second, we need to make the probability distribution of the two algorithms smoother. Therefore, after the model training, we add a temperature parameter *T* to the softmax function of trained CNN. FDFM algorithm also uses the softmax with parameter *T* to transform scores into probabilities. The softmax with the parameter *T* is described as follows:

(6) $$P\left(y=i\right)={\mathrm{Softmax}}^{T}\left(i\right)=\frac{e^{\frac{a_{i}}{T}}}{\sum_{j=1}^{C}e^{\frac{a_{j}}{T}}}$$

where P(y=i) is the possibility of the i-th categories (1≤i≤C), C is the number of categories, $a_{i}$ is the i-th value of the logits, T is the temperature parameter.

Parameter T controls the smoothness of probability distribution generated by softmax. The smaller T is, the closer the output of softmax is to one-hot code, which means the maximum value of predicted probabilities is close to 1 but the others are close to 0. If T is larger, the predicted probability distribution will be smoother. The smoothed probability distribution contributes to error correction during algorithm integration.

Following smoothing of the predicted probability distribution obtained by the two algorithms, we use D-S evidence theory to fuse the output probabilities of the two algorithms to obtain the final diagnosis results.

D-S evidence theory, first proposed by Harvard mathematician Dempster and later developed by Shafer [37], is a general framework for reasoning with uncertainty, which can be considered as a generalization of the Bayesian theory. D-S evidence theory is often used as a method of sensor fusion [38]. This theory is based on two ideas: Obtaining degrees of belief for one question from masses, and combining such degrees of belief when they are based on independent items of evidence.

In this paper, we use the fault types as frames of discernment of D-S evidence theory: $\Theta =\left\{A_{1},A_{2},\cdots ,A_{n}\right\}$ if there are n categories.$\;A_{i}$ represents the i-th fault type. Basic probability assignment (BPA), also called mass, is defined on Θ. The mass $m\left(A_{i}\right)$ of $A_{i}$ represents the degree of belief in $A_{i}$, and $m\left(A_{i}\right)$ meets the following conditions:

(7) $$m\left(\emptyset \right)=0\;0\leq m\left(A_{i}\;\right)\leq 1\;\sum_{i=1}^{n}m\left(A_{i}\;\right)=1$$

The output probabilities of the two algorithms obtained by the softmax with parameter T can be seen as the basic probability assignment function $m_{1}$ for FDFM and $m_{2}$ for CNN. Specifically, the combination, which is called the joint mass $m_{1,2}=m_{1}⨁m_{2}$, is calculated from the two sets of masses $m_{1}$ and $m_{2}$ in the following manner:

(8) $$m_{1,2}\left(A_{i}\right)=\left(m_{1}⨁m_{2}\right)\left(A_{i}\right)=\frac{m_{1}\left(A_{i}\right)m_{2}\left(A_{i}\right)}{K},i=1,2,\cdots ,n\;K=\overset{n}{\underset{i=1}{\sum }}m_{1}\left(A_{i}\right)m_{2}\left(A_{i}\right)$$

where $m_{1,2}\left(A_{i}\right)$ represents the probability that the final predicted result is $A_{i}$ after combination, K is a factor for normalization, 1−K is a measure of conflict between the two mass sets.

Finally, the combined predicted result is argmax $m_{1,2}\left(A\right)$.

## 4. Experiments

### 4.1. Data Description

**Figure 4:**
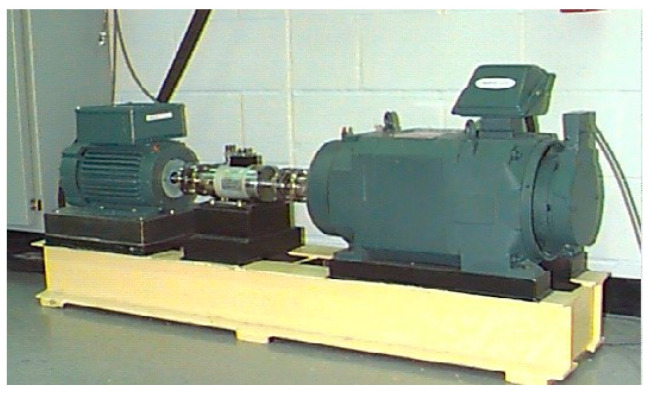
CWRU bearing experimental platform [40].

In this paper, we selected an experimental database of bearing from the Case Western Reserve University (CWRU) [39], and the sampling frequency of the dataset used for verification experiments is 12 kHz. The experimental platform is shown in Figure 4. In this experiment, rolling bearings are processed by electrical discharge machining (EDM) to simulate different fault types. The vibration signal data we analyzed in this paper are collected by the accelerators installed at the drive end.

There are four types of states: ball fault (BF), inner race fault (IRF), out race fault (ORF) and normal. Except the normal state, each fault type contains different fault diameters of 0.007 inches, 0.014 inches and 0.021 inches, so ten fault types were considered in total. The training and test samples were expanded by slicing the original vibration signal with overlap, and each obtained sample has 4096 points. For the FDFM algorithm, FFT is utilized to obtain the frequency spectrum of the sliced sample with all 4096 points. But for CNN, we only used the first 2048 points of the sliced samples for accelerating the training of CNN. Dataset A, B and C each contains 7000 training samples and 3000 test samples under loads of 1, 2 and 3 hp, which means each category contains 700 training samples and 300 test samples. The specific information of experimental samples is shown in Table 2. All experiments are based on Dataset A. Dataset B and C are used to discuss the cross-domain variation trend of frequency spectrum when the working condition changes.

**Figure 5:**
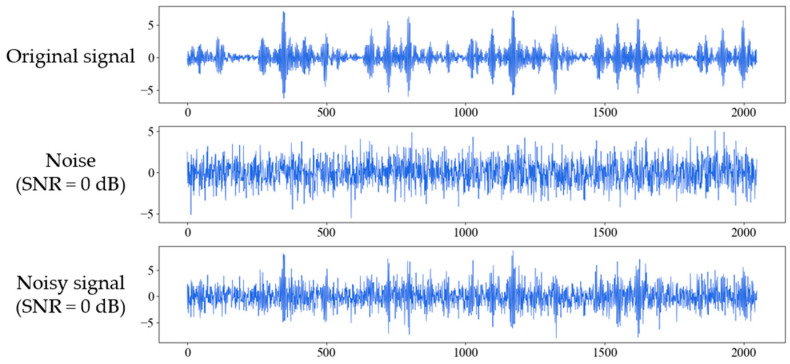
Figures for original signal of inner race fault (IRF-0.021), the additive white Gaussian noise, and the composite noisy signal with SNR = 0 dB respectively.

**Figure 6:**
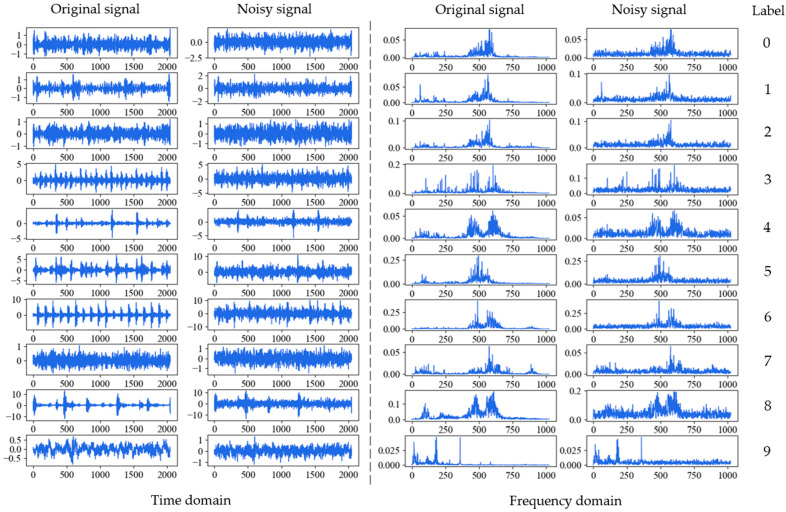
Original and noisy waveforms of the ten fault types in time-domain and frequency domain under 1 hp when SNR is 0 dB: 0: BF-0.007; 1: BF-0.014; 2: BF-0.021; 3: IRF-0.007; 4: IRF-0.014; 5: IRF-0.021; 6: ORF-0.007; 7: ORF-0.014; 8: ORF-0.021; 9: Normal.

The original dataset provided by CWRU can be considered as clean signals without noise interference, and the model proposed in this paper was trained by the original samples without noise. In order to study the robustness of the model in noise environment, we added Gaussian white noise to sliced test samples to generate noisy samples with different SNRs, and the definition of SNR is shown as follows:

(9) $$SNR=10\log_{10}\left(\frac{P_{signal}}{P_{noise}}\right)$$

where $P_{signal}$ and $P_{noise}$ are the power of signal and the noise respectively. The smaller the SNR, the greater the noise interferes with the signal. Figure 5 shows the process of adding white Gaussian noise to the original signal of inner race fault with 0.021 inches fault diameter (IRF-0.021) under 1 hp when SNR is 0 dB. Figure 6 shows the original and noisy waveforms of the ten fault types in time-domain and corresponding frequency domain under 1 hp when SNR is 0 dB.

### 4.2. Parameters Selection

#### 4.2.1. Sampling Points of FDFM

**Figure 7:**
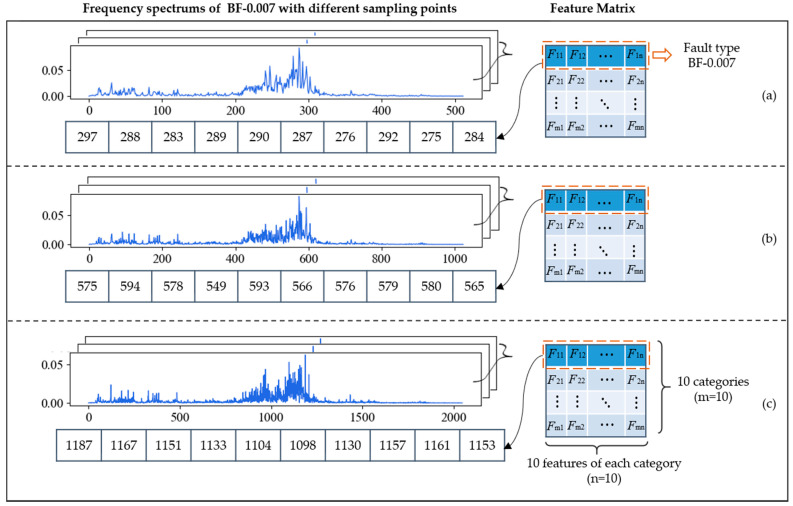
The frequency spectrums and ten feature frequencies of BF-0.007 with different sampling lengths: (**a**) 1024 points; (**b**) 2048 points; (**c**) 4096 points.

**Figure 8:**
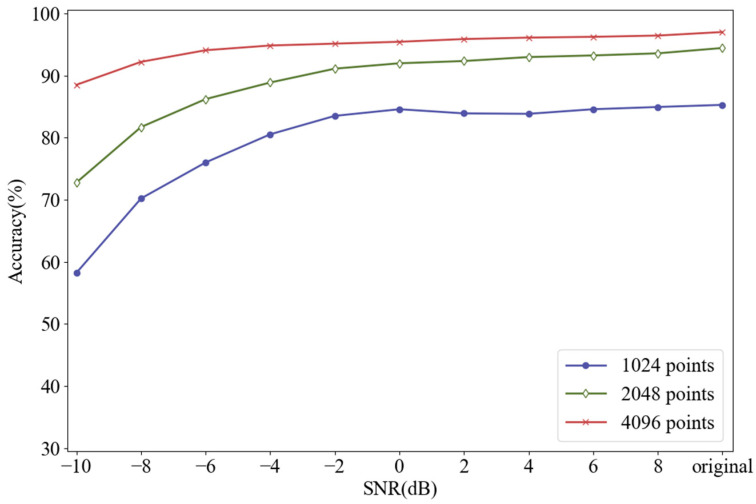
Diagnosis results of FDFM algorithm under different SNRs when the number of sampling points is 1024, 2048 and 4096 respectively.

In Section 2.1, we mentioned that increasing the number of sampling points could improve the resolution of the frequency spectrum, abscissa of which is always integer. Generally, the time domain signal with length of N can be transformed into the frequency domain signal with length of N/2 by FFT. For example, if each sliced sample has 1024 points, its frequency spectrum with the length of 512 will be obtained after FFT. Figure 7 shows the frequency spectrums of BF-0.007 with different sampling lengths. The sampling lengths of (a), (b) and (c) are 1024, 2048 and 4096, and their corresponding frequency spectrums are composed of 512, 1024, and 2048 points, respectively. At the bottom of each graph are 10 points, which represent feature frequencies of BF-0.007 obtained by FDFM algorithm, also as the first row of the feature matrix. As we mentioned in Section 2.1, the frequency of k-th point is $k\times (f_{s.max}/N)$ Hz and these points are used to represent different feature frequencies. We can see that the longer signals can generate frequency spectrum with a higher resolution by using more points, so the information in frequency domain can be expressed more completely and accurately. As sampling length increases, the measure of feature frequencies is more precise and the discrimination between adjacent points is more obvious. Figure 8 shows the diagnosis results of FDFM algorithm under different SNRs when the number of sampling points is 1024, 2048 and 4096.

**Figure 9:**
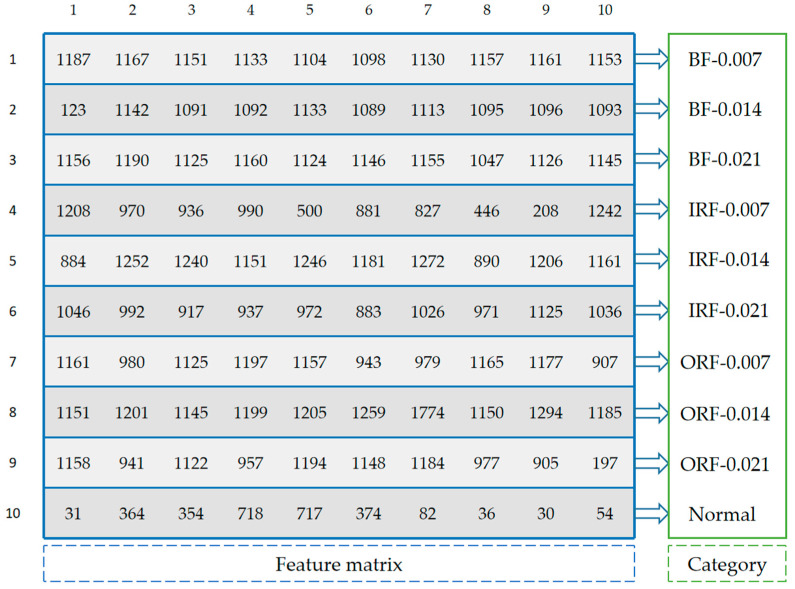
The detailed feature matrix in Figure 7c, obtained from the training of FDFM in which each sample has 4096 points.

Figure 8 shows that when the test samples are made up from the original signals, increasing the number of sampling points can improve the accuracy of FDFM algorithm. As the SNR of noisy test samples decreases, the fluctuation of accuracy is small when the sampling length is 4096. In order to express the frequency domain features more accurately and reduce the training time of the algorithm, each sample in this paper contains 4096 points. The feature matrix in Figure 7c, obtained from the training of FDFM in which each sample has 4096 points, is shown in more detail in the Figure 9.

#### 4.2.2. Scoring Rules of FDFM

**Figure 10:**
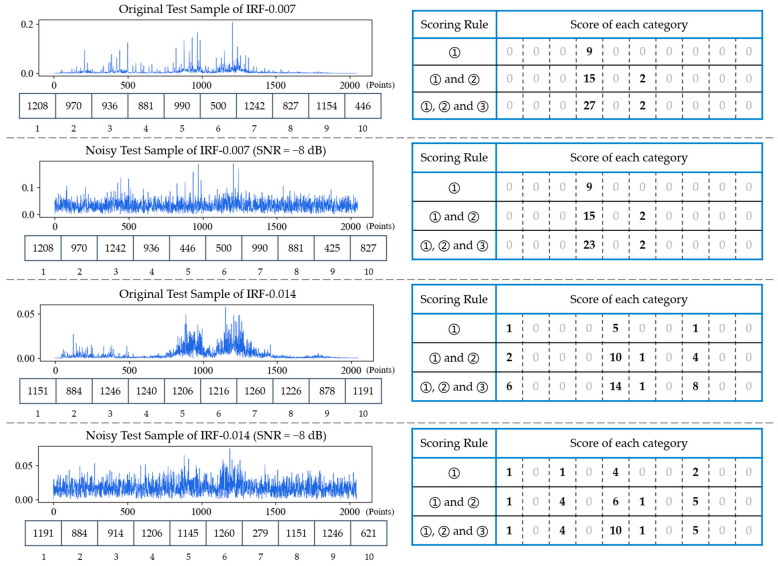
The frequency spectrums with their 10 feature frequencies of four test samples and their corresponding scoreboards obtained by different scoring rules.

In order to investigate the effectiveness of scoring rules in FDFM test stage, four test samples, each composed of 2048 points of frequency spectrum, including two original test samples of IRF-0.007, IRF-0.014 and their corresponding noisy samples (SNR = −8 dB), are selected as comparison. The results are shown in Figure 10. The FFT spectrum of each test sample and its 10 feature frequencies are on the left side. According to different scoring rules, these 10 feature frequencies are compared with each row of feature matrix in figure to obtain scoreboards, which are on the right side. It can be seen from the figure that when there is only the scoring rule (1), the scoring discrimination is not clear enough, especially in the case of noise interference. By adding the scoring rule (2) and (3), in turn, gap between the highest score and the lowest score becomes larger. Moreover, the scores of other similar categories are increased by adding (2) and (3) so that favorable error-correction information can be provided during fusion. To sum up, rule (2) can count repeatedly to increase the difference of scores, and rule (3) can increase the weight of vital feature frequencies, which are generally the frequencies of the top several peaks. Table 3 shows the diagnosis results of FDFM by different scoring rules under different SNRs. It can be seen that the accuracy increases after combination under both strong and weak noise environment. By combining these three rules, the upper limit of highest score is expanded and the scoring discrimination is much clearer, which affects the value of parameter T of softmax and provides the error-correction information during model fusion.

#### 4.2.3. First-Layer Kernel Size of CNN

**Figure 11:**
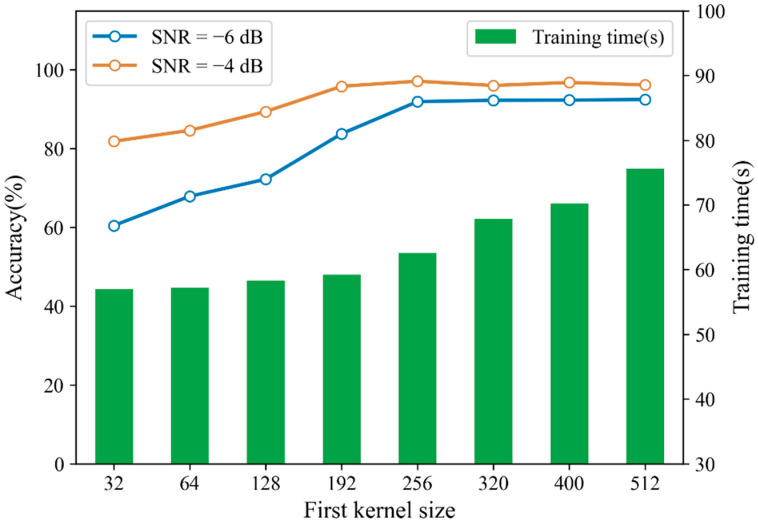
Diagnosis accuracy and training time under different sizes of first-layer convolution kernel.

In order to reduce the training time of CNN, for each sample of 4096 points, we only select the first 2048 points to train the model, and discard the other points. In Section 3.2, we mentioned that increasing the size of the first-layer convolution kernel could expand the receptive field and capture global features in a longer time domain. As described in this dataset, the minimum speed is 1730 rpm and the sampling frequency is 12 kHz, so each rotation should contain 416 sampling points. When the convolution kernel in the first layer of CNN is wider than 416, every single convolution kernel can capture the global features upon one whole period. Although increasing the size of the convolution kernel will result in a lack of some detailed features, it can reduce the dependence of the model on too subtle information in shorter time domain. When the test sample contains a large amount of noise, the short time domain signal affected by noise will reduce the diagnosis accuracy and the diagnosis of model is more dependent on the global features of the signal. Increasing the size of the first-layer convolution kernel can obtain better anti-noise performance but also increase the complexity of the model. In this experiment, we investigated diagnosis accuracy and training time of CNN with different sizes of first-layer convolution kernel. Trained CNN was tested with noisy samples with SNRs of −4 dB and −6 dB respectively. The results are shown in Figure 11. It can be seen that when the size of first-layer convolution kernel is larger than 256, the diagnosis accuracy remains relatively stable. As the size of convolution kernel continues to increase, so does the training time, while the improvement of diagnosis accuracy is insignificant. Therefore, the size of first-layer convolution kernel is selected as 256 in this paper.

#### 4.2.4. Dropout Rate

**Figure 12:**
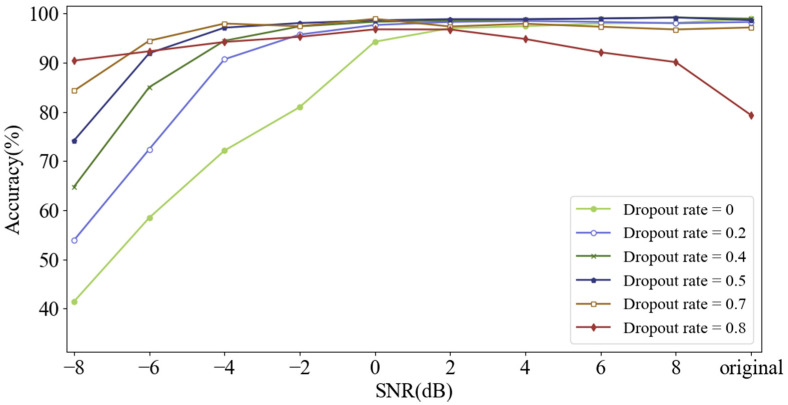
Diagnosis accuracy of CNN with different dropout rates testing on signals with different SNRs.

Dropout is used in the input layer to improve the anti-noise ability of the model. During training, the data points of the original input signal are set to zero randomly at a certain rate called dropout rate. The input signal will not be destroyed when dropout rate is set to 0. As dropout rate rises from 0 to 0.8, the noise-free training samples are destroyed excessively, which means that the proportion of destroyed data points increases. Here, the performance of CNN under different dropout rates was investigated, and the test samples were composed of noisy samples with different SNRs from −8 dB to 8 dB, as well as noise-free samples. Experimental results are shown in Figure 12.

As dropout rate increases, the accuracy under severe noise environment such as SNR of −8 dB can be improved significantly. However, as SNR increases, the diagnosis accuracy falls when model is trained with a high dropout rate such as 0.8. It can be seen that increasing dropout rate can improve the anti-noise ability of the model under severely noisy situation, but it will make diagnosis accuracy decrease in the case of weak or free noise when dropout rate is too high. Therefore, the dropout rate was determined to be a moderate value of 0.5. Meanwhile, destroyed training samples randomly generated by dropout can achieve the highest diversity when dropout rate is 0.5.

### 4.3. Performance of FDFM with Limited Sample Size

**Figure 13:**
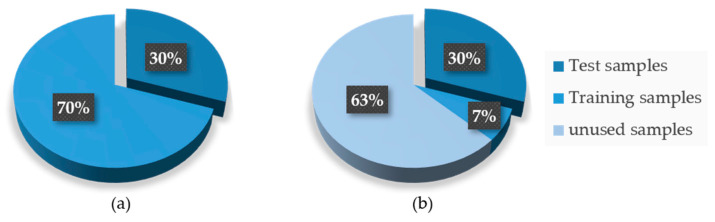
Compositions of datasets. (**a**) Original datasets; (**b**) new training datasets.

In order to study the diagnosis performance of FDFM algorithm with limited sizes of samples, five new datasets were generated by reducing the number of the training samples. Five training datasets are composed of 1%, 5%, 10%, 20% and 50% of training samples from Dataset A respectively, which means that each category of them only contains 7, 35, 70, 140, 350 training samples. Figure 13 shows how the new training dataset was composed compared to the original one.

In this experiment, 3000 test samples with SNR of −6 dB were predicted by FDFM, and the results are shown in Table 4. It can be seen that with the decrease of proportion of training samples, the diagnosis accuracy decreased slightly, but the total decrease is less than 4%. Even when the number of training samples only accounts for 1% of the original training dataset, the accuracy is still higher than 90%. As the number of training samples reduces, the training time will be greatly reduced.

The reason why the diagnosis accuracy of FDFM cannot be affected by the number of samples is that the feature matrix generated in the training stage can still be effective. Specifically speaking, the feature frequencies of the same fault type are basically consistent under the same working condition, so the feature matrix generated with few training samples can represent each fault effectively. In general, FDFM can improve the diagnosis accuracy in the case of limited sample size under noise environment, but FDFM can only be used for recognition under a single working condition. Nevertheless, it still can provide a reference to solve the problems of data scarcity and noise interference in industrial field.

### 4.4. Visualization of CNN

**Figure 14:**
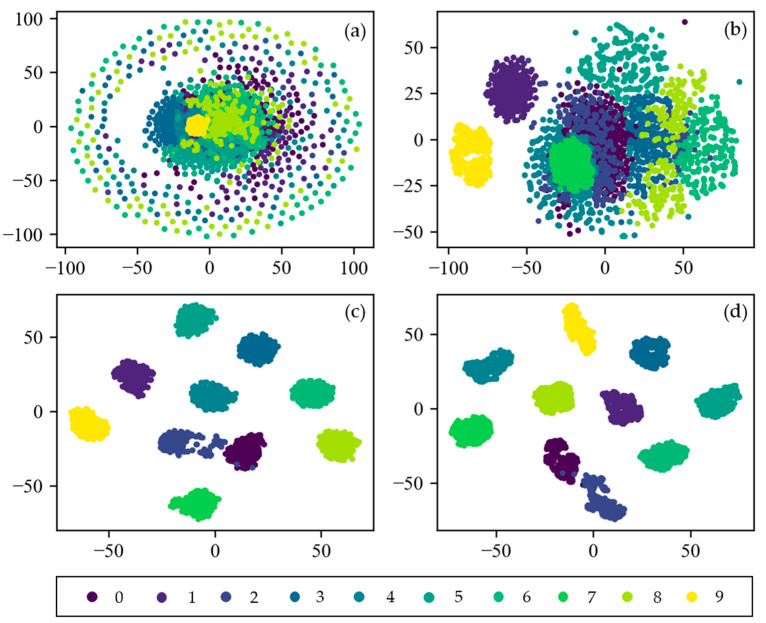
Feature visualization of CNN via t-SNE: (**a**) Raw Signal; (**b**) pooling Layer1; (**c**) pooling Layer2; (**d**) fully Connected Layer.

To visually explain the feature learning process of CNN, the t-distributed stochastic neighbor embedding (t-SNE) technique of manifold learning is applied for visualization. It can project high-dimensional data into two-dimensional or three-dimensional space, which is very suitable for visualization of high-dimensional data [41]. Figure 14 shows the feature visualization results of the input layer, the first pooling layer, the second pooling layer, and the fully connected layer of CNN. At first, the distribution of input data is so scattered that it is difficult to distinguish them. As the layers get deeper, the feature are more separable. After two layers of convolution and pooling, all 10 categories are easily distinguishable in the fully connected layer. Only the labels 0 and 2 are partially interlaced. This indicates that CNN proposed in this paper has an excellent ability in adaptive feature extraction and feature expression.

### 4.5. Model Fusion

**Figure 15:**
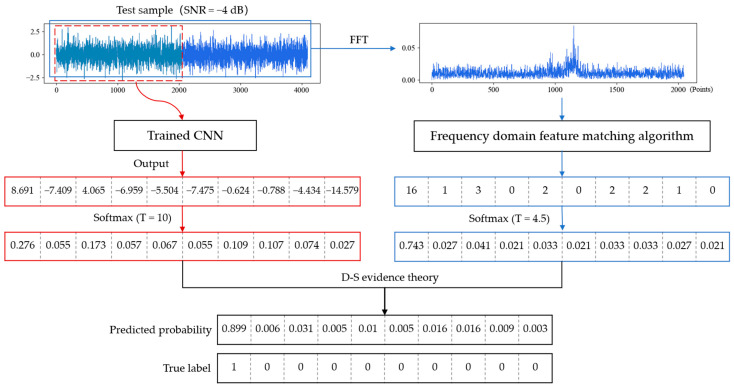
Process and result of model fusion when testing on a noisy sample with a SNR of −4 dB.

In this section, we fused the output results of the trained CNN and FDFM algorithm by D-S evidence theory. The temperature parameters of softmax in these two algorithms were determined through experiments. The parameter T was set as 10 in CNN and 4.5 in FDFM, by which the smoothed probability is conducive to fusion. Taking a test sample with a SNR of −4 dB as an example, the fusion process and results are shown in the Figure 15.

After smoothing, the highest probability is not too sharp, and some possibilities are given to the other categories. The CNN and FDFM algorithm can provide different information, so the diagnosis results are more reliable after fusion. Experiments were carried out to investigate the anti-noise performance of CNN, FDFM and their fusion model called CNN-FDFM. The models were trained with noise-free signals and tested with noisy samples with different SNRs from −10 dB to 8 dB. For each model, ten trials were carried out, and the average values were taken as the results. The specific results are shown in the Table 5.

It can be seen from the Table 5 that CNN performs well when the SNR of test samples is larger than −4 dB, and the accuracy is over 98% when SNR > 0 dB. However, as SNR decreases less than −4 dB, the accuracy falls significantly and is less than 50% when SNR is −10 dB. For FDFM, the accuracy is still high under strong noise environment, but the upper limit of accuracy is only 96~97% when SNR > 0 dB. The fusion model CNN-FDFM, which can make up for the shortcomings of both CNN and FDFM, achieves better performance and the accuracy is higher than both of CNN and FDFM after fusion. The accuracy of CNN-FDFM is over 99% when SNR is higher than −6 dB. When SNR is −10 dB, the accuracy of CNN-FDFM still reaches 93.33%, improved by 47.9% compared to CNN.

**Figure 16:**
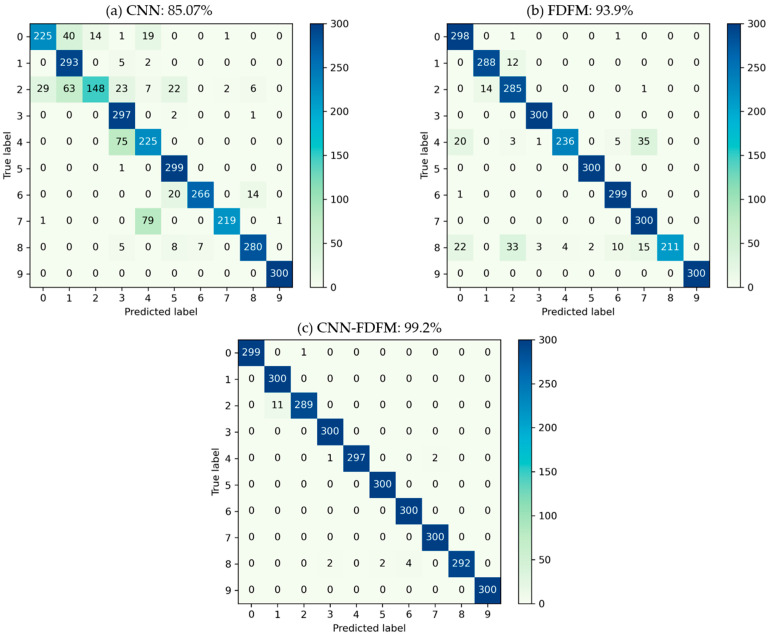
The classification confusion matrix of three models during fusion when the SNR of test samples is −6 dB: (**a**) CNN; (**b**) FDFM; (**c**) CNN-FDFM.

In order to further evaluate the classification and explain why the model performs better after fusion, confusion matrixes of CNN, FDFM and CNN-FDFM were generated. Figure 16 shows the three confusion matrixes, each of which records the diagnosis classification results when SNR is −6 dB, including both the classification information and misclassification information. The vertical axis of the confusion matrix represents the true label, and the horizontal axis represents the predicted label. Therefore, for 300 test samples of the same label, confusion matrix can show how many test samples are classified correctly and which category test samples are misclassified into. Figure 16a shows the classification results of CNN. When SNR is −6 dB, recognition of CNN is not significant upon labels 0, 2, 4 and 7. The classification results of FDFM are shown in Figure 16b. It can be seen that FDFM has poor recognition upon labels 4 and 8. The confusion matrix of CNN-FDFM is shown in Figure 16c, and the samples misclassified by CNN and FDFM are corrected to the true label mostly.

In this case, CNN-FDFM achieves better performance for two reasons: (1) When these two models recognize test samples of the same label, the accuracy of one model is high, and the accuracy of the other is relatively low. The classification results of low-precision model can be improved by high-precision model. For example, CNN is weak in recognizing samples of label 7, with only 219/300 accuracy, while the accuracy of FDFM is 300/300 under the same conditions. This indicates that FDFM can provide extra useful information to correct the samples misclassified by CNN. (2) Even though the accuracy of CNN and FDFM is not high for recognizing samples of a certain label, their misclassified categories are different. Therefore, the weight of misclassified categories can be reduced after fusion. For example, when these two models recognizing samples of label 4, the accuracy of CNN and FDFM is 225/300 and 236/300, respectively. The misclassified category of CNN is label 3 with 75 samples in it, while the misclassified categories of FDFM are label 7 with 35 samples, label 0 with 20 samples, label 2 with three samples and label 3 with one sample. Misclassified categories do not overlap, which means the predicted probability of the original misclassified categories will decrease after fusion. Therefore, the accuracy of CNN-FDFM after model fusion reaches 297/300 for recognizing 300 test samples of label 4.

### 4.6. Comparison

**Figure 17:**
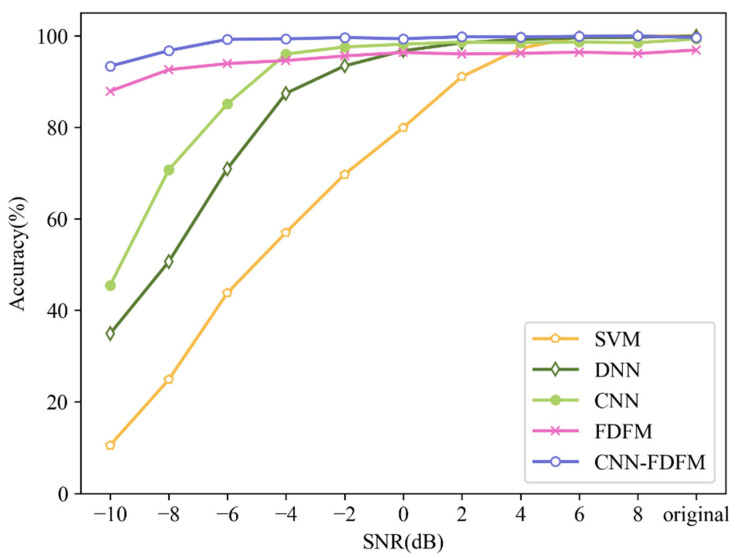
Diagnosis results of different models under different SNRs.

FDFM, CNN, CNN-FDFM, proposed in this paper and some commonly used models such as Deep Neural Network (DNN) and Support Vector Machine (SVM) are selected as comparison. The parameters of FDFM, CNN and CNN-FDFM are consistent with Section 4.5. For DNN and SVM, all samples are transformed into frequency domain by FFT, and then test samples with different SNRs are used to test the trained models. DNN has a 4-layer structure of 1024-512-256-10, and dropout is used before the last layer. The kernel function of SVM is radial basis kernel function. For each model, the average result of ten trials is used as the evaluation standard. Figure 17 shows the diagnosis results of different models under different SNRs. It can be seen that the diagnosis accuracy of each model can reach 99% except FDFM when the signals are original and noise-free. As the SNR decreases, the diagnosis accuracy of SVM falls first, followed by DNN and CNN. CNN proposed in this paper has better anti-noise ability than DNN and SVM. Besides, the upper limit of accuracy of FDFM is not high enough, no more than 97%, but the anti-noise ability of FDFM is so strong that the model after fusion also keeps this advantage. Benefiting from FDFM, the diagnosis accuracy of CNN-FDFM is 47.9% higher than CNN when SNR is −10 dB. The comparison results show that CNN-FDFM has the highest diagnosis accuracy.

To investigate the computational cost of different models with different numbers of samples, the CPU time including training time and testing time of each model is displayed in Table 6 and Table 7. All the experiments were implemented using Tensorflow toolbox of Google with an Intel i7-10700 CPU and 32G RAM. DNN, CNN and CNN-FDFM are all trained 50 epoches and batch size is 64. Training time of FDFM is the time consumption of generating the feature matrix. As shown in Table 6, when 7000 samples are used for model training, both FDFM and CNN-FDFM cost long computation time due to the complex computation of feature matrix. But when we use only 700 training samples to train the models, FDFM only costs 0.62 s for training and CNN-FDFM costs 8.23 s as Table 7 shows. Moreover, the diagnosis accuracy of FDFM and CNN-FDFM is less affected by the numbers of samples compared with DNN and CNN. In addition, the processing time for CNN-FDFM to diagnose a signal is about 1.5 ms, so CNN-FDFM can be used for real-time diagnosis.

## 5. Discussion

The anti-noise ability of model for fault diagnosis is studied in this paper. The CNN model is optimized in the time domain, and the FDFM algorithm is proposed in the frequency domain. The final diagnosis result is obtained by combining the diagnosis results of the two models. Compared with the previous studies,

(1) The anti-noise ability of our model is studied under worse noise environment. The diagnosis accuracy of some previous models decreases obviously when SNR drops to −4 dB, and most previous models are not competent for the situation where SNR is less than −4 dB. In this paper, the range of SNR was extended to −10 dB, and the accuracy was still greater than 90% when SNR is -10 dB. The comparison between some existing anti-noise models and our proposed model is shown in Table 8. All the anti-noise models were trained and tested on CWRU bearing dataset, and the diagnosis accuracy under noise environment with SNR of −4 dB was compared.

(2) The combination of time domain and frequency domain is adopted for fault diagnosis. Most of the other studies only extract fault features from one single domain for fault identification. In this paper, CNN can adaptively extract time-domain features from original signals and recognize faults automatically, which is an end-to-end model, while FDFM can extract key fault features from the frequency domain and generate feature matrix to complete fault diagnosis.

**Figure 18:**
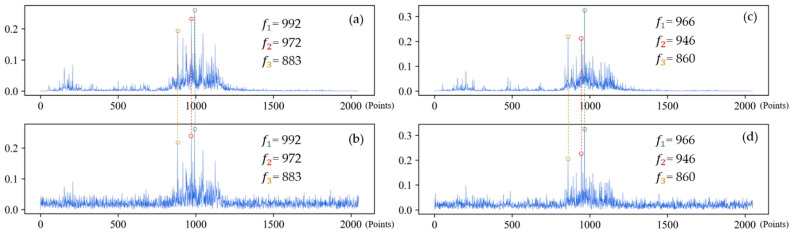
The frequency spectrums of IRF-0.021 under different working conditions. First three feature frequencies are noted in each frequency spectrum. (**a**) Frequency spectrum of original signal under 1 hp; (**b**) Frequency spectrum of noisy signal under 1 hp (SNR = 0 dB); (**c**) Frequency spectrum of original signal under 3 hp; (**d**) Frequency spectrum of noisy signal under 3 hp (SNR = 0 dB).

By the experiments in this paper, there are following findings:

(1) We confirm that the larger kernel in the first convolutional layer can make CNN achieve better performance, and the trick of dropout used in the input layer can improve the anti-noise ability of network.

(2) The results of model fusion imply that the fault information obtained from frequency domain and time domain by the two algorithms is different, but complementary to each other. Therefore, the diagnosis accuracy can be improved by information fusion and error correction. Besides, the features in frequency domain are less affected by noise.

(3) Analysis of frequency spectrum shown in Figure 18 suggests that when the sample is only affected by noise, the amplitude of frequency spectrum changes vertically, but the location of the peak frequency does not. However, when the working condition changes, the frequency spectrum shifts laterally, so does the location of the peak frequency.

## 6. Conclusions

In this paper, one-dimensional convolutional neural network fusing frequency domain feature matching algorithm named CNN-FDFM is proposed to solve the problem of strong noise interference in industry field. The analysis of experiments shows that the diagnosis accuracy of the CNN-FDFM is improved by 47.9%, compared with CNN when SNR is −10 dB. FDFM algorithm can also work in the case of limited sample size under noise environment. Novelties and contributions of this paper are summarized as follows:

(1) FDFM algorithm can learn the key features directly from the frequency domain, and solve the problem of fault identification under limited samples and strong noise interference environment.

(2) Dropout used in the first layer can simulate noise input during training of CNN. A wider kernel in the first convolutional layer can improve the anti-noise ability of CNN.

(3) Softmax with parameter T and D-S evidence theory are used to fuse different diagnosis information in time domain and frequency domain, which makes up the limitations of the two algorithms.

The model proposed in this paper has the following limitations:

(1) FDFM algorithm only pays attention to the abscissa axis of frequency spectrum, without considering the specific amplitude.

(2) FDFM algorithm is not suitable for multiple working conditions. When the working condition changes, the frequency spectrum shifts laterally and original feature matrix generated by FDFM does not work.

In view of the above limitations, further research is needed:

(1) The key features of the spectrum should be extracted intelligently and adaptively, and both the location of key features and the frequency amplitude are taken into account.

(2) To ensure the consistency of features extracted from samples under different working conditions, we can use frequency spectrums on different scales to unify features as much as possible. Moreover, rather than focusing on the specific location of peak frequencies, further studies should investigate the trend within frequency spectrum.

## Figures and Tables

**Table 1:** Structures and parameters of CNN.

Layer	Kernel Size/Step Size	Kernel Number	Output Size	Padding
Conv1	256 × 1/16 × 1	16	128 × 16	YES
Pooling1	2 × 1/2 × 1	16	64 × 16	NO
Conv2	20 × 1/5 × 1	10	9 × 10	YES
Pooling2	2 × 1/2 × 1	10	4 × 10	NO
Fully connected layer	100	1	1 × 100	
Softmax	10	1	1 × 10	

**Table 2:** Description of rolling element bearing datasets.

Fault Location		Ball			Inner Race			Outer Race			None
Category label		0	1	2	3	4	5	6	7	8	9
Fault diameter(inch)		0.007	0.014	0.021	0.007	0.014	0.021	0.007	0.014	0.021	0
Dataset Size	Train	700	700	700	700	700	700	700	700	700	700
	Test	300	300	300	300	300	300	300	300	300	300

**Table 3:** Diagnosis accuracy of FDFM by different scoring rules under different SNRs.

Accuracy (%)	SNR (dB)				
	−10	−6	−2	2	6
(1)	86.77	93.03	94.23	94.7	94.83
(1) and (2)	86.9	93.13	94.3	94.93	95.23
(1), (2) and (3)	88.73	94.47	95.2	95.9	96.37

**Table 4:** Diagnosis accuracy and training time of FDFM under different sample proportions.

Training Samples	Proportion	1%	5%	10%	20%	50%	100%
	Number	70	350	700	1400	3500	7000
Accuracy (%)		90.33	90.73	91.2	92.43	92.83	93.9
Training time (s)		0.01	0.14	0.62	2.66	19.58	91.86

**Table 5:** Diagnosis results of CNN, FDFM and CNN-FDFM under different SNRs.

Accuracy (%)	SNR (dB)										
	−10	−8	−6	−4	−2	0	2	4	6	8	Original
CNN	45.43	70.67	85.07	96	97.53	98.13	98.6	98.47	99.27	98.63	98.47
FDFM	87.77	92.57	93.9	94.57	95.57	96.33	96	96.13	96.4	96.1	96.87
CNN-FDFM	93.33	96.73	99.2	99.3	99.6	99.33	99.77	99.7	99.87	99.93	99.6

**Table 6:** The computation time of each method with 7000 training samples and 3000 test samples.

Method	Training Time (7000 Samples)	Testing Time (3000 Samples)	Accuracy (SNR = −4 dB)
DNN	39.12 s	0.125 s	87.4%
CNN	37.7 s	0.178 s	96%
FDFM	91.86 s	3.609 s	94.53%
CNN-FDFM	127.52 s	4.288 s	99.3%

**Table 7:** The computation time of each method with 700 training samples and 300 test samples.

Method	Training Time (700 Samples)	Testing Time (300 Samples)	Accuracy (SNR = −4 dB)
DNN	7.63 s	0.017 s	82.67%
CNN	7.68 s	0.065 s	86.33%
FDFM	0.62 s	0.363 s	93.33%
CNN-FDFM	8.23 s	0.477 s	98%

**Table 8:** Comparison with other anti-noise methods based on CWRU dataset.

Method	Baseline Model	Anti-Noise Strategy	Diagnosis Accuracy on CWRU Dataset (SNR = −4 dB)
WDCNN [29]	CNN	Wide kernels in the first convolutional layer	66.95%
FC-WTA [1]	SAE	Data destruction and lifetime sparsity	71.44%
TICNN [34]	CNN	Kernel with changing dropout rate and small mini-batch training	82.05%
CNN-FDFM	CNN	Anti-noise algorithm FDFM and information fusion between CNN and FDFM	99.3%

## Data Availability

Not applicable.
